# Leveraging sociological systems theory: integrating transformational leadership and social capital to enhance quality of care – an exploratory longitudinal study of German hospitals

**DOI:** 10.1186/s12913-026-15268-6

**Published:** 2026-08-06

**Authors:** Arno Stöcker, Jeffrey Braithwaite, Ludwig Kuntz, Verena Maschke, Holger Pfaff

**Affiliations:** 1 Institute of Medical Sociology, Health Services Research and Rehabilitation Science, Faculty of Medicine and University Hospital Cologne, University of Cologne, Cologne, Germany; 2 Centre for Health Services Research Cologne, Interfaculty Institution of the University of Cologne, Cologne, Germany; 3 Center for Health Services Research and Communication, Department for Psychosomatic Medicine and Psychotherapy, University Hospital Bonn and Faculty of Medicine, University of Bonn, Venusberg- Campus 1, Bonn, 53127 Germany; 4 Australian Institute of Health Innovation, Macquarie University, Sydney, Australia; 5 Department of Business Administration and Health Care Management, Faculty of Management, Economics and Social Sciences, University of Cologne, Cologne, Germany; 6 Department of Cardiology, Faculty of Medicine and University Hospital Cologne, University of Cologne, Cologne, Germany

**Keywords:** Patient safety, Senior medical leader, Learning organization, Healthcare management, Organizational behavior, Social systems theory

## Abstract

**Background:**

Numerous studies have examined ways to improve care quality and patient safety. Further progress could be achieved by leveraging underexplored sociological concepts. To date, transformational leadership and social capital have been examined primarily as isolated factors in healthcare research to explain variations in hospital care quality. Yet, these concepts have rarely been integrated into a broader theoretical framework. Drawing on Parsons’ AGIL framework, we identified two key social prerequisites for effective healthcare organizations: goal attainment and integration. We hypothesized that hospitals meeting both criteria would deliver better care quality.

**Methods:**

Using a 2008 survey of medical directors in German hospitals, we measured transformational leadership (as a proxy for goal attainment) and social capital (as a proxy for integration). Data were matched with quality metrics from nationwide reports (2012–2019) on quality irregularities and quality deficiencies. Hospitals were grouped into high and low categories for both factors using a median split, and longitudinal panel analyses were conducted on 508 hospitals.

**Results:**

The combination of strong transformational leadership and high social capital was associated with significantly fewer quality irregularities, but not with quality deficiencies.

**Conclusions:**

Our findings suggest that hospital leadership should strategically strengthen both factors to improve organizational capacity for quality improvement. This synergy likely fosters goal-oriented collective action, particularly the collaborative efforts required to enhance care quality and patient safety. Additionally, we propose that applying sociological theory offers valuable insights into the social dynamics essential for maintaining and enhancing quality and safety in healthcare settings.

## Background

 Ensuring the quality of care and promoting patient safety stand as pivotal objectives within healthcare [1]. Although some interventions have proven effective [2, 3], the broader literature indicates that system-wide improvements in quality of care have remained limited [4, 5]. These limitations are attributed both to the modest overall gains reported in the field [4, 5] and to the difficulty of establishing clear empirical links between specific quality-improvement interventions and demonstrable enhancements in care and patient safety [6].

A possible explanation for this phenomenon is the ongoing challenge in deeply comprehending the intricate dynamics of quality and patient safety, coupled with the requisite contextual conditions [7], essential for the effective implementation of corresponding measures. In response, the research community has intensified empirical efforts to enhance this understanding [6, 8]. Additionally, an emerging perspective emphasizes the need to complement this empirical approach with a theory-based approach in quality and safety [9, 10]. As a consequence, more initiatives have focused on developing new theories or frameworks through qualitative research [11], psychological and social-psychological approaches [12], and safety-specific theoretical models (e.g., normal accident theory, high reliability organization theory) [13].

Although research on quality and safety often seeks theoretical foundations, sociological theories have been surprisingly underutilized [14]. This is noteworthy because theory-based interventions, such as those designed to promote behavior change, have been shown to be more effective [15]. This raises concerns, considering that healthcare organizations operate as social systems [16], where quality initiatives emerge from collective learning and action, transcending individual efforts [4, 5]. Recognizing this gap, commendable efforts have recently emerged to integrate sociological theoretical approaches into research on quality and safety [17, 18]. We endorse the ongoing exploration of this promising stance and seek to contribute to its advancement.

### Systemic challenges in the German healthcare context

German hospitals operate in a highly regulated, decentralized system shaped by self-governed organizations of providers and payers, a dual financing structure (statutory health insurance and federal states), a reimbursement system based on diagnosis related groups, a fragmented governance landscape, and growing obligatory quality assurance [19]. They exhibit a rigid and standardized organizational structure, featuring a uniform top management team composed of a medical director, a nursing director, and a commercial director, alongside highly compartmentalized clinical departments. Each department is headed by a clinical director who serves as the lead physician, holds ultimate responsibility for clinical decisions, and acts as the superior to all physicians within the department [20]. In recent years a focus on quality, cost-effectiveness, and the integration of the in- and outpatient sector governs the transformation efforts in the inpatient sector [21], though these structural changes are described as uncoordinated [22] and barriers remain between the integration of in- and outpatient care [23].

The inpatient sector faces challenges due to conflicting values and norms between staff and the organization, as well as cultural differences across professional groups, generations, and personal backgrounds. These tensions can hinder effective collaboration and integration [24]. The leadership structure in a hospital is described as crucial for the organizational performance [25] with a lack of appreciation for commitment, work, and performance among employees [26] and insufficient coordination and communication [23] as main challenges in German hospitals.

### Sociological perspective on quality of care and patient safety

In essence, sociology can contribute to the exploration of quality and safety in healthcare in three key ways. The first addresses the macro level within organizations, aiming to clarify the role of context, structures, and system features in ensuring and enhancing quality and safety [14]. The second focuses on the micro level, shedding light on the role of interactions, collective practices, and processes of sense-making [27]. The third seeks to illuminate the interplay between the macro and micro levels [28, 29] in the generation of quality and safety. While the second and third forms of contribution are well suited for delineating and analyzing processes of improvement, change, and implementation in quality and safety, the first is instrumental in scrutinizing the impact of organizational structures on the quality of care.

Our objective is to contribute to the first perspective by examining whether specific social structural features within hospitals have a lasting impact on quality and safety. Safety researchers emphasize the significance of system factors in quality and safety [3, 5, 30]. Thus, it is surprising that systems theory in general, including established social systems theories, has not been widely applied to safety analysis and improvement. We firmly believe that among the wide array of sociological theories, sociological system theories hold particular promise in providing fresh insights into the systemic factors influencing quality and safety.

In this context, we contend that Talcott Parsons’ structural–functional theory is especially valuable for analyzing the fundamental prerequisites of quality and safety in hospitals [4, 7, 11]. Parsons’ AGIL scheme [31] specifies four core functions that any social system must fulfil to generate reliable outcomes over time: adaptation to its environment, goal attainment, integration to ensure stability, and latency to preserve core values. Applied to inpatient care, this means that a hospital must secure and adapt resources and competencies to shifting clinical and regulatory environments (A), define, prioritize and pursue goals such as high quality of care and patient safety (G), integrate and coordinate its professional groups and units to ensure coherent action (I), and sustain shared norms and institutionalized values that support a robust safety culture (L).

Collectively, these functions provide a theoretically grounded lens for examining how structural and cultural conditions shape organizational performance in the domain of quality and safety. Within this analytical framework, the functions of goal attainment and integration constitute particularly consequential prerequisites for coordinated quality and safety efforts, and we elaborate on these in the following section.

#### Goal attainment and integration as prerequisites for successful collective action in healthcare organizations

The quality of healthcare is significantly influenced by effective cooperation and coordination among hospital departments, health professionals, and individuals [1]. This enterprise hinges on the healthcare organization’s overarching ability to consistently establish and pursue goals, and on its general ability to foster cohesive collective action. Two conditions of Parsons’ AGIL scheme become instrumental here: goal attainment and integration. These prerequisites for collective quality actions are met when there is a prevailing pattern of goal attainment behavior within the healthcare organization (G function) and when the network of employees is socially integrated (I function). Healthcare organizations have inherent characteristics that make coordination challenging. They function as professional bureaucracies [32], multicultural entities [33], highly specialized organizations [34], and are affected by interprofessional conflicts [35] and tensions between professionals and management [36]. These factors make it difficult to set common goals [37], achieve social integration and cohesion [38, 39], and maintain sufficient collaboration [40]. Goal-oriented collective action, in general, and collective safety action, in particular, are facilitated by the general ability to set and pursue goals (G), to integrate the group of professionals (I), and to align the group towards these goals.

We understand transformational leadership (TL) as a suitable proxy for goal attainment, as it fosters motivation, aligns group objectives, and mobilizes individuals toward collective aims. Social capital (SC) effectively captures the integrative function by reflecting the strength of social ties, trust, and shared norms that enable cohesion and cooperation within a system.

#### Transformational leadership and goal attainment

Goal setting, particularly when coupled with feedback for goal attainment, consistently leads to enhanced overall performance [3]. It correlates with improved quality and safety behavior [37], contributes to a more robust safety culture [41], and yields superior safety outcomes [2]. Furthermore, TL behavior, as a distinctive leadership style within a hospital’s leadership team, promotes collaboration in setting and achieving goals with followers [42] and has been linked with modest yet notable improvements in work and task performance [43]. Significantly, TL has been demonstrated to enhance safety behavior of workers [44], cultivate a positive safety climate [45], and improve patient safety outcomes [46].

TL in German hospitals has been the focus of various studies, covering several key areas. Research has examined the impact of TL on the relational quality between executive staff and different professional groups [47], as well as interventions designed to promote transformational leadership and well-being among hospital leaders from diverse disciplines [48]. Other studies have explored the potential of TL among nurse leaders [49], its role in improving patient safety climate, occupational safety climate, and overall working conditions in German university hospitals [50], and its association with the frequency of incident reporting [51]. Further findings suggest a link between TL and occupational safety culture [52] and its relevance in addressing generation-specific challenges in nursing management [57]. Additionally, TL has been shown to promote interprofessional collaboration and support broader system-wide change processes [53]. It remains a prominent topic in the German healthcare discourse, especially in relation to its potential contribution to addressing the ongoing nursing care crisis [24].

#### Social capital and integration

An integrated collective of professionals is characterized by high SC and can be regarded as a collaborative community [54]. Studies have established a positive relationship between SC and quality of care [39]. Additionally, studies have shown that good collaboration between professionals, one of the results of SC, enhances quality and safety outcomes [55], though on a limited evidence base [40].

SC in the context of German hospitals has been examined from various perspectives. Research has explored aspects such as coordination among hospital staff [56], the relationship between SC and outcomes like burnout, well-being, and work engagement among hospital employees [38], as well as its positive influence on network culture within inter-hospital trauma center networks [57]. Additional studies have investigated SC among physicians in German breast cancer centers in relation to patient-physician interactions [58], its impact on physician job satisfaction [59], the link between organizational SC and emotional exhaustion in hospitals [60], and its role in promoting work engagement among physicians [61].

#### Combining goal attainment and integration: guiding the collective energy of an integrated group with a purpose

In the pursuit of ensuring and elevating quality and safety, the synergy of goal attainment and integration becomes paramount, forming what we term the GI factor. The full potential of the GI factor is realized, for instance, when leaders align organizational goals with the objectives of the cohesive collectivity they lead [62]. It is also evident when a unified collective strives for shared, self-determined, or co-created goals [63]. In both scenarios, a dynamic interplay between goal-orientation and social integration unfolds, guiding the collective energy of the cohesive group. The collective energy of a cohesive group is given a direction by leaders or the group itself. This can be accomplished through the group´s active participation in goal-setting and the leader´s ability to persuade and inspire the group toward organizational objectives. Successful fusion of G and I leads to further interactions, where joint goal-setting contributes to integration [41] and social integration facilitates collaborative decision-making. In advance to this study, the combined influence of social capital and transformational leadership on clinical risk management in German hospitals has been studied [64].

Hence, we hypothesize that the co-occurrence of goal attainment behavior in leaders (G), measured through TL, and the social integration of health care professionals (I), measured by SC, serves as a predictor of quality and safety outcomes in hospitals.

## Methods

We deployed a longitudinal study design. As the main explanatory variable (GI factor) is time-invariant, we deployed a panel regression model with between estimators. We used data from two sources. First, information on the quality of care, patient safety, and general organizational data of hospitals was derived from the national-wide mandatory quality reports of German hospitals across the years 2012–2019. Second, data on TL (proxy for goal attainment) and SC (proxy for integration) came from a 2008 survey of medical directors of German hospitals.

### Data source 1: quality reports of German hospitals

German hospitals are obliged to collect and publish a quality report annually. These reports form a fundamental pillar of transparency in the inpatient sector in Germany [22]. Data are collected and publicly accessible, published in accordance with Sects. 136 and 137 of the German Social Security Code V. The reports provide a comprehensive insight into the structures, services, and quality of the respective hospitals and their specialist departments. In these annual reports, hospitals document their performance and organizational metrics as well as various quality indicators and scores at the department level. If previously defined reference values are exceeded, a structured dialogue is initiated with the respective hospital. A structured dialogue aims to assess whether arithmetically conspicuous results (outside of the reference ranges) are “qualitatively conspicuous” [65] and clarifies why they have occurred [66].

Since 2005, all hospitals have been obliged by law to prepare and publish quality reports. Initially, these reports were compiled and published every two years. Since 2012, this reporting has been conducted annually. Furthermore, from 2012 onward, a new system for evaluating the results of quality indicators and conspicuousness criteria was established [67]. Thus, we have chosen the period from 2012 to 2019, the last year before the COVID-19 pandemic, since the measures taken in the course of the pandemic have significantly influenced everyday hospital practice [68] and the process of compiling and publishing the quality report [69].

#### Outcome variable: classification of quality indicators

Within the quality reports, quality indicators are grouped into seven assessment categories (Table 1). For our analysis, we omitted all quality indicators where a valuation was not intended (category *N*). In addition, we assigned the remaining six categories into three groups:

1. *no quality issues*: quality indicators with results within the reference range (category *R*) and quality indicators that have undergone the process of a structured dialogue due to conspicuous results but for which the evaluation established qualitative inconspicuous results (category *U*);

2. *quality irregularities*: quality indicators that have undergone the process of a structured dialogue and where some sort of quality irregularities was detected (categories *H*, *D*, *S*);

3. *quality deficiencies*: quality indicators for which a quality deficiency was identified during the structured dialogue (category *A*).

In general, quality irregularities (such as documentation and software problems, notifications of hospitals without clarification of the relevance of the irregularity, and other reasons) are not as severe for the quality of care and patient safety as detected quality deficiencies [70]. Nevertheless, we contend that they signify non-optimal organizational features related to quality and safety management topics. Quality irregularities can be understood as early warning indicators for the potential occurrence of quality deficiencies, reflecting the collective attitude toward quality and safety management. Undoubtedly, they highlight imperfections in the organization’s procedures and processes. In contrast, quality deficiencies are indicative of problems related to health outcomes.

From our assignment, we formed two separate outcome variables for each hospital: (1) share of quality indicators with quality irregularities and (2) share of quality indicators with quality deficiencies. The denominator for both groups was the total number of all quality indicators for which a valuation was intended. During the study period, the absolute number of quality indicators per hospital declined steadily. The focus shifted from purely descriptive quality indicators to indicators with quality targets and reference categories to analyze the quality of healthcare and patient safety relevant to patients and hospital management [65].

**Table 1:** Categorization of quality indicators. Depiction according to IQTIG (based on IQTIG. Bericht zum Strukturierten Dialog 2020. Berlin 2021 [70]; own translation)

Category	Classification	Number	Explanatory note	Authors’ classification for analysis
N	Valuation not intended	01	Quality indicator without result, as no corresponding cases have occurred.	*Omitted from analysis*
		02	Reference range is not defined for this indicator.	
		99	Other (explained in the comments)	
R	Result within reference range	10	Result is computationally inconspicuous; no structured dialogue is necessary.	No quality issues detected
U	Evaluation according to structured dialogue as qualitative inconspicuous	30	Correct documentation is confirmed (data validation)	
		31	Special clinical situation.	
		32	The deviating result is explained by individual cases.	
		33	No evidence of deficiencies in medical quality (isolated documentation problems).	
		99	Other (explained in the comments)	
H	Institution notified of arithmetically conspicuous result	20	Request for internal quality management to analyse the computational abnormality.	Quality irregularities detected
		99	Other (explained in the comments)	
D	Evaluation not possible because of faulty documentation	50	Incomplete or incorrect documentation.	
		51	Software problems have caused incorrect documentation.	
		99	Other (explained in the comments)	
S	Other	90	Waiver of measures in structured dialogue.	
		91	Structured dialogue not yet completed.	
		99	Other (explained in the comments)	
A	Evaluation according to structured dialogue as qualitative conspicuous	40	Incorrect documentation is confirmed (data validation)	Quality deficiencies detected
		41	Indications of structural and process deficiencies.	
		42	No (sufficiently explanatory) reasons for the computational abnormality have been identified.	
		99	Other (explained in the comments)	

### Data source 2: survey on transformational leadership and social capital

Data on SC and TL were obtained from the ATräk study (Auswirkungen unterschiedlicher Trägerstrukturen von Krankenhäusern auf die Qualität der Krankenversorgung der Bevölkerung [Effects of differing hospital ownership structures on the quality of healthcare for the population]) on effects of hospital ownership structures on quality of healthcare in 2008 [56]. Medical directors were selected as suitable survey subjects due to their strategic and unique perspectives on organizational and clinical expertise as key informants [64]. Furthermore, they influence and assess processes, structures, and organizational culture [64, 71].

In the survey, SC was measured by a validated scale (Social Capital of Organisations [SOCAPO]; [38]) with six aspects of the overall construct: mutual understanding, trust, warm circle, mutual help, we-feeling, and common values. A Likert scale was used for the responses, ranging from “strongly disagree” (1) to “strongly agree” (4). TL was surveyed by a six-item scale based on six key behaviors of transformational leaders identified by Podsakoff et al. [72]. The medical directors were asked to assess how often the executives of their hospital, such as top management, head physicians, and nursing management, exhibited transformational behavior. A Likert scale was used ranging from “never” (1) to “always” (5).

### Explanatory variable: GI factor

For our analysis, we dichotomized both scales using a median split technique; thus, two levels of the GI factor were generated (Table 2): High GI level (high SC and high TL), and low GI level (low SC and/or low TL). As we argue that both parts of the GI factor must be present, we refrained from solely multiplying both scales. Through multiplication, a high value in one scale would compensate for a large deficiency in the other, contradicting our theoretical derivation of the GI factor.

**Table 2:** The goal-integration factor: goal-oriented integrated collective (based on Pfaff H, Braithwaite J. 2020 [64])

	Integration = Low (SC< median [SC])	Integration=High (SC > = median [SC])
Goal attainment=High(TL< median [TL])	Goal-oriented individuals	Goal-oriented cohesive collective (GI factor)
Goal attainment = Low (TL > = median [TL])	Non-goal-oriented individuals	Non-goal-oriented cohesive collective

### Statistical analysis

We employed a panel data model with a between estimator as the main explanatory variable (GI factor) remained invariant over time. The organizational variables ownership (public, non-profit, private), number of beds (per 100), and teaching status (no teaching assignment, academic teaching hospital, university hospital) were included as control variables. We developed three models for each of the two outcome variables: (1) a base model including all control variables; (2) a model adding the GI factor to assess its potential association with the outcome variables; and (3) a model incorporating the GI factor along with TL and SC to examine whether the association of the GI factor persists when these components are modeled separately. A sustained effect of the GI factor in the third model would suggest that its association is not fully accounted for by TL and SC alone, indicating an interactive rather than purely additive relationship. Because TL and SC were measured in 2008 while quality indicator data were collected between 2012 and 2019, our models assume relative stability of these constructs over time. Potential changes in hospital leadership, policies, or integration practices could affect this assumption; further discussion is provided in the Limitations section.

Data preparation and analysis were performed with the R software (version 4.3.2), RStudio (version 2023.12.0), and the following packages: tidyverse (2.0.0), plm (2.6-3), clubsandwich (0.5.10), modelsummary (1.4.5), ggthemes (5.1.0), and gtsummary (1.7.2).

### Data analysis

From the 551 hospitals of the original ATräk study, 22 were excluded as they did not publish any quality reports in the period 2012–2019, and 21 were excluded because they submitted quality reports for multiple sites of one hospital. Additionally, some hospitals did not publish a report every year. Consequently, the final dataset was unbalanced. Overall, data on 508 hospitals with 3,940 observations were included in the study population, representing roughly one-third of all German hospitals.

## Results

### Description of the study population

A total of 205 hospitals had a high GI factor, while 303 hospitals had a low GI factor. The mean TL score was 3.6 (median 3.7), while the mean SC score was 2.9 (median 3) (Table 3). A sharp decrease in the average number of quality indicators per hospital can be noted, from 161.4 in 2012 to 81.8 in 2019. Especially from 2015 on, a sharp decline can be seen. On average, 77.9 quality indicators were classified as evaluable by the performing authorities. Over time, the share of evaluated quality indicators increased from 68.6% in 2012 to 84.9% in 2019. On average, 8.1 quality indicators (10.4%) resulted in a structured dialogue per hospital annually. The absolute number of structured dialogues decreased over time (2012: 10.9 to 2019: 6.9), while the relative number or share of structured dialogues remained constant. Furthermore, the absolute number of detected quality irregularities decreased over time (2012: 6.0 to 2019: 3.3), while the relative number or share of quality irregularities slightly decreased (2012: 5.4% to 2019: 4.8%). Finally, the absolute number of detected quality deficiencies remained constant over time (2012: 1.2 to 2019: 1.1); accordingly, the relative number or share increased (2012: 1.1% to 2019: 1.5%).

**Table 3:** Descriptive table of study population (*N* = 3,940, *n* = 508)

Characteristic	Overall, *N* = 3,940 (*n* = 508)^1^	2012, *N* = 489^1^	2015, *N* = 494^1^	2019, *N* = 483^1^
**GI-factor**				
low	2,350 (59.6%)	291 (59.5%)	295 (59.7%)	288 (59.6%)
high	1,590 (40.4%)	198 (40.5%)	199 (40.3%)	195 (40.4%)
**Transformational leadership**	3.6 / 3.7 (0.6)			
**Social capital**	2.9 / 3.0 (0.5)			
**Number of beds**	335.7 / 258.0 (281.7)	332.9 / 253.0 (277.5)	336.4 / 258.0 (280.4)	337.8 / 260.0 (287.6)
**Ownership**				
non-profit	1,757 (44.6%)	217 (44.4%)	223 (45.1%)	211 (43.7%)
private	545 (13.8%)	67 (13.7%)	66 (13.4%)	70 (14.5%)
public	1,638 (41.6%)	205 (41.9%)	205 (41.5%)	202 (41.8%)
**Teaching status**				
no teaching assignment	1,628 (41.3%)	227 (46.4%)	199 (40.3%)	185 (38.3%)
academic teaching hospital	2,150 (54.6%)	245 (50.1%)	277 (56.1%)	274 (56.7%)
university hospital	162 (4.1%)	17 (3.5%)	18 (3.6%)	24 (4.8%)
**Number of quality indicators**	107.7 / 99.0 (50.1)	161.4 / 165.0 (49.2)	84.9 / 85.5 (39.4)	81.8 / 81.0 (31.4)
**Number of quality indicators** (without quality indicators no evaluation intended)	77.9 / 75.0 (34.6)	110.8 / 111.0 (38.2)	53.3 / 53.0 (24.9)	69.5 / 68.0 (28.3)
**Structured dialogues**	8.1 / 7.0 (5.4)	10.9 / 10.0 (6.7)	6.2 / 6.0 (4.4)	6.9 / 6.00(4.9)
**Detected quality irregularities**	4.0 / 3.0 (3.5)	6.0 / 5.0 (4.8)	3.0 / 2.0 (2.7)	3.3 / 2.0 (3.3)
**Detected quality deficiencies**	1.1 / 1.0 (1.5)	1.2 / 1.0 (1.6)	1.0 / 0.0 (1.4)	1.1 / 1.0 (1.5)

**1 Mean / Median (SD); n (%)**

### Visualization of the trend over time

**Fig. 1:**
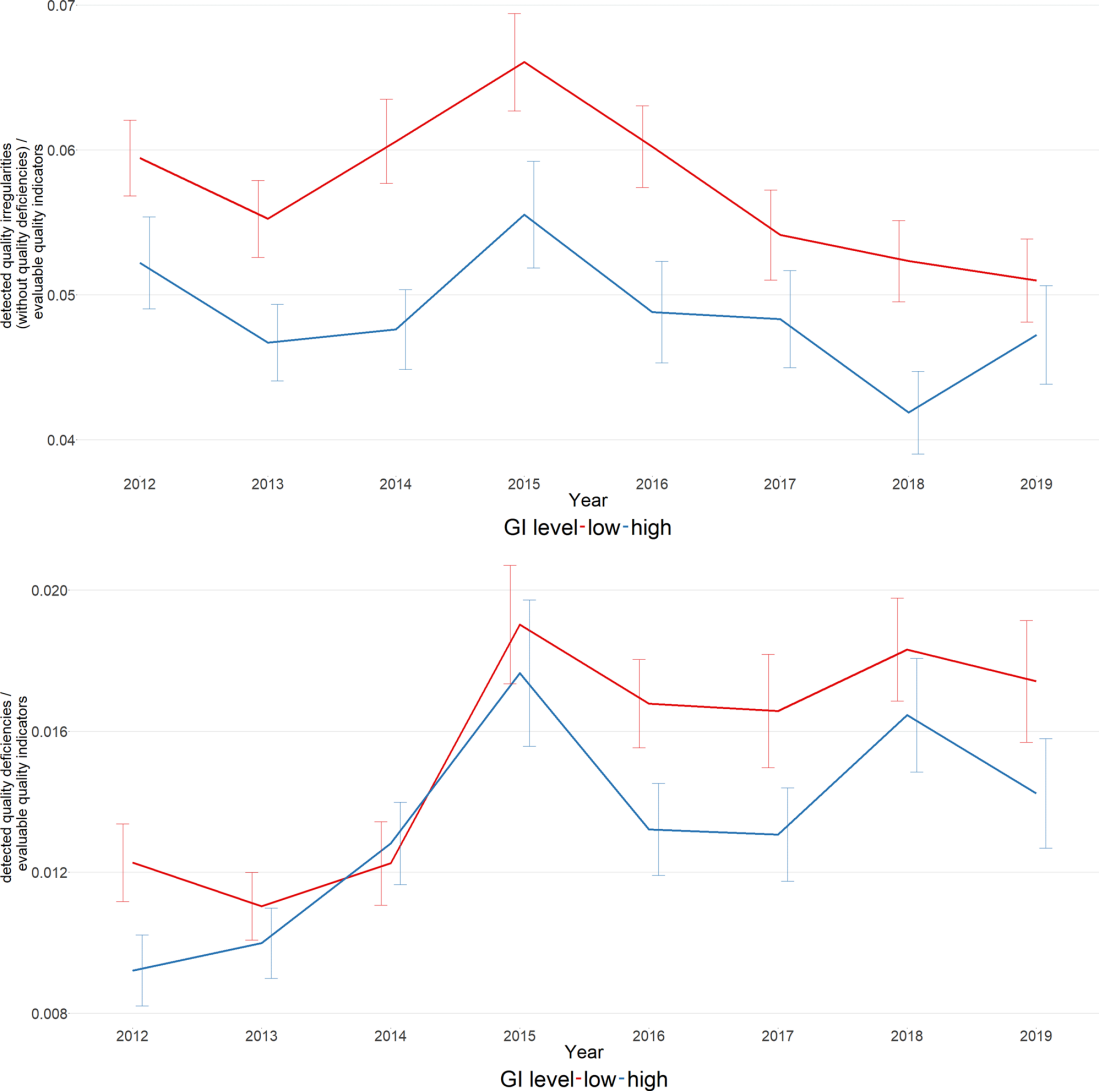
Year-by-year (2012–2019) share of detected quality irregularities (quality indicator classifications H, D, and S) with CI (upper chart) and share of quality deficiencies (quality indicator classification A) with CI (lower chart) by all evaluable quality indicators

We first visualized the two outcome variables (share of quality irregularities and share of quality deficiencies) with respect to the GI factor (Fig. 1). Overall, the share of quality irregularities decreased, although the trend fluctuated over time. Hospitals with a low GI level showed a higher share of quality irregularities than hospitals with a high GI factor. The confidence intervals overlapped only in 2017 and 2019.

For the second examination, the graphical visualization was similar to that of the previous examination. Overall, the trend that hospitals with a high GI factor had a lower share of quality deficiencies persisted for all years except 2014, though the difference between the GI levels was less distinct and more profound in the latter years. The confidence intervals were non-overlapping only in the years 2012, 2016, and 2017.

### Panel model with a between-effects estimator

We constructed six panel models with between-effects estimators to analyze the effect of the GI factor variable on the share of quality irregularities and deficiencies on all evaluable quality indicators per hospital (Table 4). First, a base model for quality irregularities was constructed (Model 1) with the controlling variables ownership, number of beds (per 100), teaching status, TL, and SC. Both TL and SC showed no significant associations. The R² for the model was 0.085. From this base model, we added the GI factor variable to Model 2. Hospitals with a high GI level had a significantly lower share of quality indicators with reported quality irregularities (-0.009, *p* = .001). Compared to the base model, the R² rose to 0.097. In the third Model, we controlled for SC and TL. The negative association (-0.011) for the GI factor remained significant (*p* = .009). 

**Table 4:** Linear regression models (between-effects estimator) for the GI factor on the share of quality irregularities and quality deficiencies (n = 508)

	Model I: Quality irregularities (base model)	Model II: Quality irregularities (GI model)	Model III: Quality irregularities (GI model controlled)	Model IV: Quality deficiencies (base model)	Model V: Quality deficiencies (GI model)	Model VI: Quality deficiencies (GI model controlled)
**Intercept**	0.085***	0.068***	0.063***	0.023***	0.015***	0.021***
95%-CI	[0.064, 0.105]	[0.062, 0.074]	[0.036, 0.089]	[0.015, 0.030]	[0.013, 0.017]	[0.012, 0.031]
SE	(0.011)	(0.003)	(0.013)	(0.004)	(0.001)	(0.005)
p-value	(< 0.001)	(< 0.001)	(< 0.001)	(< 0.001)	(< 0.001)	(< 0.001)
**GI-factor high (RC: GI-factor low)**		-0.009**	-0.011**		-0.002*	-0.001
95%-CI		**[-0.015**,** -0.004]**	**[-0.019**,** -0.003]**		**[-0.004**,** 0.000]**	[-0.004, 0.002]
SE		**(0.003)**	**(0.004)**		**(0.001)**	(0.001)
p-value		**(0.001)**	**(0.009)**		**(0.046)**	(0.637)
**Ownership: public (RC: non-profit)**	0.007*	0.007*	0.007*	0.002	0.002	0.002
95%-CI	**[0.001**,** 0.014]**	**[0.001**,** 0.014]**	**[0.001**,** 0.014]**	[-0.001, 0.004]	[-0.001, 0.004]	[-0.001, 0.004]
SE	**(0.003)**	**(0.003)**	**(0.003)**	(0.001)	(0.001)	(0.001)
p-value	**(0.020)**	**(0.020)**	**(0.020)**	(0.189)	(0.178)	(0.190)
**Ownership: private (RC: non-profit)**	-0.001	-0.001	-0.001	0.002	0.002	0.002
95%-CI	[-0.010, 0.008]	[-0.010, 0.007]	[-0.010, 0.007]	[-0.002, 0.005]	[-0.001, 0.005]	[-0.002, 0.005]
SE	(0.004)	(0.004)	(0.004)	(0.002)	(0.002)	(0.002)
p-value	(0.820)	(0.773)	(0.754)	(0.309)	(0.296)	(0.317)
**Number of beds (by 100)**	-0.003***	-0.003***	-0.003***	0.000	0.000	0.000
95%-CI	**[-0.004**,** -0.002]**	**[-0.004**,** -0.002]**	**[-0.004**,** -0.002]**	[-0.001, 0.000]	[-0.001, 0.000]	[-0.001, 0.000]
SE	**(0.001)**	**(0.001)**	**(0.001)**	(0.000)	(0.000)	(0.000)
p-value	**(< 0.001)**	**(< 0.001)**	**(< 0.001)**	(0.750)	(0.800)	(0.744)
**Teaching status: academic teaching hospital (RC: no teaching assignment)**	-0.007*	-0.007*	-0.007*	-0.001	-0.001	-0.001
95%-CI	**[-0.014**,** 0.000]**	**[-0.014**,** 0.000]**	**[-0.014**,** 0.000]**	[-0.004, 0.001]	[-0.004, 0.001]	[-0.004, 0.001]
SE	**(0.004)**	**(0.004)**	**(0.004)**	(0.001)	(0.001)	(0.001)
p-value	**(0.037)**	**(0.040)**	**(0.040)**	(0.263)	(0.261)	(0.266)
**Teaching status: university hospital (RC: no teaching assignment)**	0.018+	0.019+	0.020+	0.010**	0.010**	0.010**
95%-CI	[-0.001, 0.038]	[0.000, 0.039]	[0.000, 0.039]	**[0.002**,** 0.017]**	**[0.003**,** 0.017]**	**[0.002**,** 0.017]**
SE	(0.010)	(0.010)	(0.010)	**(0.004)**	**(0.004)**	**(0.004)**
p-value	(0.068)	(0.052)	(0.050)	**(0.009)**	**(0.008)**	**(0.009)**
**Transformational leadership**	-0.002		0.002	-0.001		-0.001
95%-CI	[-0.009, 0.004]		[-0.005, 0.009]	[-0.003, 0.001]		[-0.003, 0.002]
SE	(0.003)		(0.004)	(0.001)		(0.001)
p-value	(0.446)		(0.617)	(0.410)		(0.602)
**Social capital**	-0.004		0.000	-0.002		-0.002
95%-CI	[-0.011, 0.003]		[-0.008, 0.008]	[-0.004, 0.001]		[-0.004, 0.001]
SE	(0.004)		(0.004)	(0.001)		(0.001)
p-value	(0.283)		(0.984)	(0.194)		(0.304)
Num.Obs.	508	508	508	508	508	508
R2	0.085	0.097	0.098	0.047	0.044	0.048
R2 Adj.	0.072	0.086	0.083	0.034	0.033	0.033
AIC	-2056.9	-2065.6	-2061.8	-3076.6	-3076.9	-3074.8
BIC	-2018.8	-2031.7	-2019.5	-3038.5	-3043.0	-3032.5

+ p < 0.1, * p < 0.05, ** p < 0.01, *** p < 0.001

For the next three models, we changed the outcome variable. In Model 4, the base model for quality deficiencies, both TL and SC showed no significant associations. The R² was 0.047. Again, in Model 5, with the GI factor incorporated, it was apparent that a high GI level was associated with a lower share of quality deficiencies (-0.002; *p* = .046). The R² was 0.044. When controlled for SC and TL (Model 6) the association no longer remained significant.

Models estimated using random-effects and correlated random-effects approaches yielded similar effect sizes. The GI factor showed significant associations with quality irregularities both with and without the inclusion of TL and SC (Appendix Table  1), and significant associations with quality deficiencies only in the model excluding TL and SC (Appendix Table 2). Additionally, we constructed models in which the GI factor was categorized into terciles (Appendix Table 3) and quintiles (Appendix Table 4). Although the direction of the association remained consistent, no significant correlations were found between the GI factor and quality irregularities. For quality deficiencies, a significant association was observed in models excluding TL and SC; however, this effect was no longer significant once TL and SC were included. Validation analysis provides evidence that Category H (‘Institution notified of arithmetically conspicuous result’) was primarily responsible for the significant associations found in relation to quality irregularities as analysis without that category did not show significant associations for the GI factor (Appendix Table 5).

## Discussion

This exploratory study aimed to illustrate how system theories can be operationalized and empirically test. The analysis revealed significant differences in the quality of care and patient safety across German hospitals, with those possessing a high TL and SC (GI factor) displaying significantly lower shares of quality irregularities. When assessing the implications and impact of the results, it is essential to consider the low occurrence of quality irregularities. While the descriptive proportion of quality irregularities was 4%, the model estimates a baseline level of 6.8%. Based on this, the explanatory variable was associated with a 0.9% point reduction, corresponding to a relative effect of about 13%.

For quality deficiencies, the analysis found no significant difference. Overall, quality deficiencies are rare for German hospitals, making it difficult to achieve adequate statistical power for any explanatory variable. The result may be interpreted in two ways: either German hospitals generally provide sufficient quality of care, or the quality indicators used fail to adequately capture quality deficiencies. The latter interpretation is supported by recurring reports of quality concerns in German hospitals [73, 74] suggesting that current quality measurement tools may be inadequate [75].

The former interpretation - that German hospitals provide generally sufficient quality of care - is also plausible. Hospitals have frequently been described as high-reliability organizations [76], a trait that can be extended to the healthcare system in Germany. Consequently, it is not surprising that quality deficiencies are relatively infrequent. In Germany, outcome quality is assured through extensive formal training for medical staff, abundant resources, and a comprehensive set of guidelines and standards. However, process quality is more likely shaped by organizational factors. This suggests that the GI factor serves as a more effective tool for assessing quality processes than for evaluating quality and safety outcomes.

Robustness checks yield a more nuanced picture. Although the direction of the associations remained consistent across alternative model specifications the strength and significance of these relationships varied. This suggests that the association between the GI factor and quality deficiencies may be fully accounted for by the additive contributions of TL and SC, rather than reflecting a distinct synergistic effect. Dichotomizing both measures resulted in an information loss, and the selection of the median split is arbitrarily chosen with a relative rather than an absolute cut-off limit. Additionally, the lack of significance in the tercile and quintile models suggests that the association may be sensitive to how the GI factor is operationalized.

Taken together, these findings indicate that while the GI factor appears to capture meaningful organizational conditions related to quality performance, particularly for quality irregularities, its explanatory power may be more limited or context-dependent when it comes to quality deficiencies. These inconsistencies highlight the need for cautious interpretation.

Recognizing healthcare as a complex environment [77], our research indicates that the influence of the GI factor is not a standalone construct for achieving better quality and patient safety. Other aspects influencing patient safety include patient engagement, human resources management quality, innovation technology, skills certification, education in patient safety, teamwork, and effective communication [78].

Interventions to foster TL are common in health care settings [79]. A wide range of TL intervention programs exists; however, most consist of single training sessions, making outcome comparisons difficult, [79]. Research suggests that TL can be effectively cultivated through structured leadership development programs focused on emotional intelligence, communication skills, and empowering team dynamics [80, 81]. Effective measures to enhance TL include tailored programs that combine group-based workshops with individualized feedback, action planning, and coaching. Emphasizing core behaviors like individualized consideration, intellectual stimulation, fostering group goals, and setting high performance expectations has been shown to improve leader behavior, team cohesion, and organizational outcomes [79]. TL programs show better outcomes when developed together with the specific target population [79].

Results for the enhancing of SC from intervention studies in the health care setting are limited [82]. Hospitals can build staff SC through measures such as collaborative leadership and flattened hierarchies promoting mutual respect and trust [57, 83]. Structured team interventions (e.g. interprofessional rounds and daily safety huddles) empower staff to share concerns and improve communication [84, 85]. Facilitating regular social events, mentoring of new personnel, and selective hiring for teamwork skills further strengthens interpersonal ties [38].

Finally, we outlined a pathway for empirically applying sociological systems theories in quality and safety research. This was an exploratory approach. The concepts of social capital and transformational leadership utilized here should be perceived as an example of two functions in Parson’s AGIL schemes (especially goal attainment and integration). Despite the choice of SC and TL as proxies for integration and goal attainment, other concepts may be adoptable too; e.g. for SC social cohesion or institutional trust, and for TL transactional leadership or strategic goal orientation. The AGIL schema itself is a system-theoretical model that is considerably more comprehensive. In this work, only two aspects were adopted which themselves are considerably more extensive.

### Research strengths and limitations

We derived our data from two independent sources and, thus, avoided a common method bias for this analysis. Nonetheless, the survey itself on social capital and transformational leadership was based on one-person’s judgment and opinion, the medical chief director of the hospital. Thus, we cannot provide an objective or multi-perspective assessment of both concepts but must rely on our subjects’ judgements, which is a potential bias and may or may not align with the perspectives of other senior management staff in the hospital. We see medical chief directors as uniquely positioned to provide insights on aspects of organizational culture as they operate at the intersection of clinical practice and organizational management in German hospitals. Their dual role enables them to perceive and influence team dynamics, build trust-based networks, and drive strategic vision within the organization. However, including the perspective of other chief management staff such as chief nursing officer would have enhanced the validity and depth of the findings, as it would have captured diverse perspectives on interpersonal trust, collaboration, and leadership dynamics across professional boundaries.

Furthermore, as the survey participation in the ATräk-study was voluntary, we cannot rule out a selection bias. The measurements of the concepts of SC and TL, though validated, are open to possible forms of bias, but both provide satisfactory reliability [64]. While the assessment of SC and TL was subjective, our outcome variables were objective in nature, as they derived from an intensive quality process within the German inpatient sector. This enhances the robustness of our results, highlighting the significance of the GI concept in the realm of healthcare quality.

The analysis spans longitudinally, a departure from the prevalent cross-sectional approaches often seen in health and medical social research. The survey on SC and TL was conducted in 2008, four years prior to the start of the study period. It is not apparent how both constructs have developed since then. Therefore, our study is based on the explicit assumption of organizational inertia, in which organizational cultures change only slowly and possible changes are random across hospitals. We are confident of the validity of this assumption as success rates for culture change are low [86] and changes are challenging for organizations and their staff [71]. Hence, organizational culture can be regarded in some respects as rather time constant. However, some studies described a shift in organizational cultures in hospitals over time [87, 88]. Thus, we cannot rule out changes in both concepts in the observed hospitals and possible effects that come along with those shifts. The survey focused on medical directors’ assessments of the overall hospital climate and leadership behavior of executives, indicating that these organizational aspects can be considered to be more stable over time. Unlike the personal leadership style of a medical director, which may be subject to change with personnel shifts, the organizational culture and behavior of medical and nursing executives are perceived as less susceptible to change. However, as before, this is an assumption that we cannot support with empirical data. Hence, further longitudinal studies need to monitor aspects of organizational culture regularly throughout the study period.

Furthermore, as the survey and assessment methods for the quality indicators differed between 2008 and 2012, no analysis of the differences between the assessments of the quality indicators could be constructed and performed.

A key limitation of this study concerns potential endogeneity arising from unobserved or unmodelled factors. While we used a between-effects estimator to examine long-term associations across hospitals, this approach is essentially a cross-sectional analysis of unit-level averages and does not account for time-invariant unobserved heterogeneity. As a result, the estimated relationships may be biased due to omitted variables or reverse causality. For instance, unmeasured organizational characteristics (such as leadership turnover, strategic priorities, or regional health policy influences) may simultaneously affect both the explanatory variables (e.g. transformational leadership and social capital) and the quality outcomes. Although our primary goal was to identify structural associations rather than make causal claims, future research should explore methods suited to causal inference, such as fixed-effects models, instrumental variables, or longitudinal designs that capture within-unit variation over time.

## Conclusions

The combined presence of transformational leadership and social capital was associated with a significantly higher quality of care and enhanced patient safety in German hospitals. These findings empirically support elements of Parsons’ AGIL framework. Hospitals that incorporated a combination of goal attainment and integration (GI factor) exhibited significantly fewer quality irregularities over an eight-year period. Our findings provide evidence that successful quality and safety management necessitates two critical components: leaders who pursue clear goals and successfully align their teams with these objectives, and a cohesive, well-integrated organizational culture. Leadership development programs that emphasize goal setting, vision, and team alignment while also fostering trust, collaboration, and shared norms among staff can offer valuable benefits for hospital administrators.

## Data Availability

The ATräk datasets used and/or analysed during the current study are available from the corresponding author on reasonable request. Quality reports are publicly available from the Gemeinsamer Bundesausschuss (Federal Joint Committee) on request.

## Appendix 1

**Table:** Linear regression models (correlated-random-effects estimator) for of the GI factor on the share of structured dialogues and quality deficiencies (n = 508)

	Model I:Quality irregularities (base model)	Model II:Quality irregularities (GI model)	Model III:Quality irregularities (GI model controlled)	Model IV:Quality deficiencies (base model)	Model V:Quality deficiencies (GI model)	Model VI:Quality deficiencies (GI model controlled)
Intercept	0.080***	0.064***	0.060***	0.022***	0.014***	0.021***
95%-CI	[0.060, 0.100]	[0.058, 0.070]	[0.034, 0.086]	[0.014, 0.029]	[0.012, 0.016]	[0.011, 0.030]
SE	(0.010)	(0.003)	(0.013)	(0.004)	(0.001)	(0.005)
p-value	(<0.001)	(<0.001)	(<0.001)	(<0.001)	(<0.001)	(<0.001)
GI-factor high (Ref. Category: GI-factor low)		-0.009**	-0.010*		-0.002+	-0.001
95%-CI		[-0.014, -0.003]	[-0.018, -0.002]		[-0.004, 0.000]	[-0.004, 0.002]
SE		(0.003)	(0.004)		(0.001)	(0.002)
p-value		(0.001)	(0.014)		(0.053)	(0.681)
Ownership: public (Ref. Category: Ownership non-profit)	0.008**	0.008**	0.008**	0.003*	0.003*	0.003*
95%-CI	[0.002, 0.014]	[0.002, 0.013]	[0.002, 0.014]	[0.000, 0.005]	[0.000, 0.005]	[0.000, 0.005]
SE	(0.003)	(0.003)	(0.003)	(0.001)	(0.001)	(0.001)
p-value	(0.006)	(0.006)	(0.006)	(0.025)	(0.023)	(0.025)
Ownership: private (Ref. Category: Ownership non-profit)	0.003	0.003	0.003	0.002	0.002	0.002
95%-CI	[-0.005, 0.011]	[-0.005, 0.011]	[-0.005, 0.011]	[-0.001, 0.005]	[-0.001, 0.005]	[-0.001, 0.005]
SE	(0.004)	(0.004)	(0.004)	(0.002)	(0.002)	(0.002)
p-value	(0.478)	(0.514)	(0.521)	(0.232)	(0.224)	(0.236)
Number of beds (by 100)	-0.002**	-0.002**	-0.002**	0.000	0.000	-0.000
95%-CI	[-0.003, -0.001]	[-0.003, -0.001]	[-0.003, -0.001]	[-0.000, 0.000]	[-0.000, 0.000]	[-0.000, 0.000]
SE	(0.001)	(0.001)	(0.001)	(0.000)	(0.000)	(0.000)
p-value	(0.002)	(0.001)	(0.001)	(0.996)	(0.948)	(1.000)
Teaching status: academic teaching hospital (Ref. Category: Teaching status: no teaching assignment)	-0.007**	-0.007**	-0.007**	-0.001	-0.001	-0.001
95%-CI	[-0.012, -0.002]	[-0.012, -0.002]	[-0.012, -0.002]	[-0.003, 0.001]	[-0.003, 0.001]	[-0.003, 0.001]
SE	(0.002)	(0.002)	(0.002)	(0.001)	(0.001)	(0.001)
p-value	(0.003)	(0.003)	(0.003)	(0.484)	(0.478)	(0.487)
Teaching status: university hospital (Ref. Category: Teaching status: no teaching assignment)	-0.003	-0.002	-0.002	0.006*	0.006*	0.006*
95%-CI	[-0.016, 0.011]	[-0.015, 0.011]	[-0.015, 0.011]	[0.000, 0.012]	[0.000, 0.012]	[0.000, 0.012]
SE	(0.007)	(0.007)	(0.007)	(0.003)	(0.003)	(0.003)
p-value	(0.689)	(0.760)	(0.764)	(0.043)	(0.039)	(0.042)
Transformational leadership	-0.003		0.001	-0.001		-0.001
95%-CI	[-0.009, 0.003]		[-0.006, 0.008]	[-0.003, 0.001]		[-0.003, 0.002]
SE	(0.003)		(0.003)	(0.001)		(0.001)
p-value	(0.369)		(0.753)	(0.345)		(0.513)
Social capital	-0.003		0.000	-0.002		-0.001
95%-CI	[-0.011, 0.004]		[-0.008, 0.008]	[-0.004, 0.001]		[-0.004, 0.002]
SE	(0.004)		(0.004)	(0.001)		(0.001)
p-value	(0.358)		(0.961)	(0.258)		(0.366)
Num.Obs.	3939	3939	3939	3939	3939	3939
R2	0.012	0.014	0.014	0.005	0.004	0.005
R2 Adj.	0.011	0.013	0.012	0.003	0.003	0.003
AIC	-14479.6	-14480.4	-14478.3	-19406.4	-19407.4	-19405.4
BIC	-14423.1	-14430.2	-14415.5	-19349.9	-19357.2	-19342.6

+ p < 0.1, * p < 0.05, ** p < 0.01, *** p < 0.001

## Appendix 2

**Table:** Linear regression models (correlated-random-effects estimator) for of the GI factor on the share of structured dialogues and quality deficiencies (n = 508)

	Model I: Quality irregularities (base model)	Model II: Quality irregularities (GI model)	Model III: Quality irregularities (GI model controlled)	Model IV: Quality deficiencies (base model)	Model V: Quality deficiencies (GI model)	Model VI: Quality deficiencies (GI model controlled)
Intercept	0.082***	0.065***	0.061***	0.022***	0.014***	0.021***
95%-CI	[0.062, 0.102]	[0.059, 0.071]	[0.036, 0.087]	[0.015, 0.030]	[0.012, 0.017]	[0.011, 0.031]
SE	(0.010)	(0.003)	(0.013)	(0.004)	(0.001)	(0.005)
p-value	(< 0.001)	(< 0.001)	(< 0.001)	(< 0.001)	(< 0.001)	(< 0.001)
**GI-factor high (Ref. Category: GI-factor low)**		**-0.009****	**-0.010***		-0.002+	-0.001
95%-CI		**[-0.015**,** -0.004]**	**[-0.018**,** -0.002]**		[-0.004, 0.000]	[-0.004, 0.002]
SE		**(0.003)**	**(0.004)**		(0.001)	(0.002)
p-value		**(0.001)**	**(0.014)**		(0.051)	(0.682)
**Ownership: public (Ref. Category: Ownership non-profit)**	**0.008****	**0.008****	**0.008****	**0.003***	**0.003***	**0.003***
95%-CI	**[0.003**,** 0.014]**	**[0.003**,** 0.014]**	**[0.003**,** 0.014]**	**[0.000**,** 0.005]**	**[0.000**,** 0.005]**	**[0.000**,** 0.005]**
SE	**(0.003)**	**(0.003)**	**(0.003)**	**(0.001)**	**(0.001)**	**(0.001)**
p-value	**(0.005)**	**(0.004)**	**(0.004)**	**(0.022)**	**(0.021)**	**(0.023)**
**Ownership: private (Ref. Category: Ownership non-profit)**	0.003	0.002	0.002	0.002	0.002	0.002
95%-CI	[-0.005, 0.011]	[-0.005, 0.010]	[-0.006, 0.010]	[-0.001, 0.005]	[-0.001, 0.005]	[-0.001, 0.005]
SE	(0.004)	(0.004)	(0.004)	(0.002)	(0.002)	(0.002)
p-value	(0.517)	(0.550)	(0.562)	(0.243)	(0.233)	(0.247)
**Number of beds (by 100)**	0.002	0.002	0.002	0.001	0.001	0.001
95%-CI	[-0.001, 0.005]	[-0.001, 0.005]	[-0.001, 0.005]	[-0.001, 0.003]	[-0.001, 0.003]	[-0.001, 0.003]
SE	(0.002)	(0.002)	(0.002)	(0.001)	(0.001)	(0.001)
p-value	(0.256)	(0.259)	(0.260)	(0.192)	(0.194)	(0.192)
**Mean number of beds (by 100)**	**-0.004***	**-0.004***	**-0.004***	-0.001	-0.001	-0.001
95%-CI	**[-0.008**,** -0.001]**	**[-0.008**,** -0.001]**	**[-0.008**,** -0.001]**	[-0.003, 0.001]	[-0.003, 0.001]	[-0.003, 0.001]
SE	**(0.002)**	**(0.002)**	**(0.002)**	(0.001)	(0.001)	(0.001)
p-value	**(0.021)**	**(0.021)**	**(0.021)**	(0.178)	(0.186)	(0.178)
**Teaching status: academic teaching hospital (Ref. Category: Teaching status: no teaching assignment)**	**-0.007****	**-0.007****	**-0.007****	-0.001	-0.001	-0.001
95%-CI	**[-0.011**,** -0.002]**	**[-0.011**,** -0.002]**	**[-0.011**,** -0.002]**	[-0.003, 0.001]	[-0.003, 0.001]	[-0.003, 0.001]
SE	**(0.002)**	**(0.002)**	**(0.002)**	(0.001)	(0.001)	(0.001)
p-value	**(0.006)**	**(0.006)**	**(0.006)**	(0.550)	(0.543)	(0.554)
**Teaching status: university hospital (Ref. Category: Teaching status: no teaching assignment)**	-0.001	-0.000	-0.000	**0.006***	**0.006***	**0.006***
95%-CI	[-0.014, 0.012]	[-0.013, 0.013]	[-0.013, 0.013]	**[0.001**,** 0.012]**	**[0.001**,** 0.012]**	**[0.001**,** 0.012]**
SE	(0.007)	(0.007)	(0.007)	**(0.003)**	**(0.003)**	**(0.003)**
p-value	(0.903)	(0.976)	(0.982)	(0.032)	(0.029)	(0.031)
**Transformational leadership**	-0.003		0.001	-0.001		-0.001
95%-CI	[-0.009, 0.004]		[-0.006, 0.008]	[-0.003, 0.001]		[-0.003, 0.002]
SE	(0.003)		(0.003)	(0.001)		(0.001)
p-value	(0.402)		(0.713)	(0.357)		(0.526)
**Social capital**	-0.004		-0.000	-0.002		-0.001
95%-CI	[-0.011, 0.003]		[-0.008, 0.008]	[-0.004, 0.001]		[-0.004, 0.002]
SE	(0.004)		(0.004)	(0.001)		(0.001)
p-value	(0.308)		(0.963)	(0.241)		(0.346)
Num.Obs.	3939	3939	3939	3939	3939	3939
R2	0.014	0.015	0.015	0.005	0.005	0.005
R2 Adj.	0.012	0.014	0.013	0.003	0.003	0.003
AIC	-14483.9	-14484.6	-14482.5	-19407.2	-19408.1	-19406.2
BIC	-14421.1	-14428.1	-14413.5	-19344.4	-19351.6	-19337.1

+ p < 0.1, * p < 0.05, ** p < 0.01, *** p < 0.001

## Appendix 3

**Table:** Linear regression models (between-effects estimator) for terciles of the GI factor on the share of structured dialogues and quality deficiencies (n = 508)

	Model I:Quality irregularities (base model)	Model II:Quality irregularities (GI model)	Model III:Quality irregularities (GI model controlled)	Model IV:Quality deficiencies (base model)	Model V:Quality deficiencies (GI model)	Model VI:Quality deficiencies (GI model controlled)
Intercept	0.085***	0.065***	0.087***	0.023***	0.015***	0.020***
95%-CI	[0.064, 0.105]	[0.059, 0.071]	[0.063, 0.112]	[0.015, 0.030]	[0.012, 0.017]	[0.011, 0.029]
SE	(0.011)	(0.003)	(0.013)	(0.004)	(0.001)	(0.005)
p-value	(<0.001)	(<0.001)	(<0.001)	(<0.001)	(<0.001)	(<0.001)
GI-factor high (Ref. Category: GI-factor low)		-0.003	0.002		-0.003*	-0.002
95%-CI		[-0.011, 0.004]	[-0.007, 0.011]		[-0.006, -0.000]	[-0.005, 0.002]
SE		(0.004)	(0.005)		(0.001)	(0.002)
p-value		(0.350)	(0.697)		(0.021)	(0.277)
Ownership: public (Ref. Category: Ownership non-profit)	0.007*	0.007*	0.007*	0.002	0.001	0.001
95%-CI	[0.001, 0.014]	[0.001, 0.014]	[0.001, 0.014]	[-0.001, 0.004]	[-0.001, 0.004]	[-0.001, 0.004]
SE	(0.003)	(0.003)	(0.003)	(0.001)	(0.001)	(0.001)
p-value	(0.020)	(0.020)	(0.020)	(0.189)	(0.209)	(0.209)
Ownership: private (Ref. Category: Ownership non-profit)	-0.001	-0.001	-0.001	0.002	0.002	0.002
95%-CI	[-0.010, 0.008]	[-0.009, 0.008]	[-0.010, 0.008]	[-0.002, 0.005]	[-0.001, 0.005]	[-0.002, 0.005]
SE	(0.004)	(0.004)	(0.004)	(0.002)	(0.002)	(0.002)
p-value	(0.820)	(0.878)	(0.819)	(0.309)	(0.278)	(0.307)
Number of beds (by 100)	-0.003***	-0.003***	-0.003***	-0.000	-0.000	-0.000
95%-CI	[-0.004, -0.002]	[-0.004, -0.002]	[-0.004, -0.002]	[-0.001, 0.000]	[-0.001, 0.000]	[-0.001, 0.000]
SE	(0.001)	(0.001)	(0.001)	(0.000)	(0.000)	(0.000)
p-value	(<0.001)	(<0.001)	(<0.001)	(0.750)	(0.837)	(0.768)
Teaching status: academic teaching hospital (Ref. Category: Teaching status: no teaching assignment)	-0.007*	-0.007*	-0.007*	-0.001	-0.001	-0.001
95%-CI	[-0.014, -0.000]	[-0.014, -0.000]	[-0.014, -0.000]	[-0.004, 0.001]	[-0.004, 0.001]	[-0.004, 0.001]
SE	(0.004)	(0.004)	(0.004)	(0.001)	(0.001)	(0.001)
p-value	(0.037)	(0.038)	(0.036)	(0.263)	(0.296)	(0.290)
Teaching status: university hospital (Ref. Category: Teaching status: no teaching assignment)	0.018+	0.018+	0.018+	0.010**	0.010**	0.010**
95%-CI	[-0.001, 0.038]	[-0.001, 0.038]	[-0.001, 0.038]	[0.002, 0.017]	[0.003, 0.017]	[0.002, 0.017]
SE	(0.010)	(0.010)	(0.010)	(0.004)	(0.004)	(0.004)
p-value	(0.068)	(0.068)	(0.070)	(0.009)	(0.008)	(0.009)
Transformational leadership	-0.002		-0.003	-0.001		-0.001
95%-CI	[-0.009, 0.004]		[-0.009, 0.004]	[-0.003, 0.001]		[-0.003, 0.002]
SE	(0.003)		(0.003)	(0.001)		(0.001)
p-value	(0.446)		(0.400)	(0.410)		(0.638)
Social capital	-0.004		-0.005	-0.002		-0.001
95%-CI	[-0.011, 0.003]		[-0.012, 0.003]	[-0.004, 0.001]		[-0.004, 0.002]
SE	(0.004)		(0.004)	(0.001)		(0.001)
p-value	(0.283)		(0.254)	(0.194)		(0.409)
Num.Obs.	508	508	508	508	508	508
R2	0.085	0.079	0.085	0.047	0.047	0.050
R2 Adj.	0.072	0.068	0.071	0.034	0.035	0.034
AIC	-2056.9	-2055.5	-2055.0	-3076.6	-3078.2	-3075.8
BIC	-2018.8	-2021.6	-2012.7	-3038.5	-3044.4	-3033.5

+ p < 0.1, * p < 0.05, ** p < 0.01, *** p < 0.001

## Appendix 4

**Table:** Linear regression models (between-effects estimator) for quintiles of the GI factor on the share of structured dialogues and quality deficiencies (n = 508)

	Model I:Quality irregularities (base model)	Model II:Quality irregularities (GI model)	Model III:Quality irregularities (GI model controlled)	Model IV:Quality deficiencies (base model)	Model V:Quality deficiencies (GI model)	Model VI:Quality deficiencies (GI model controlled)
Intercept	0.085***	0.065***	0.086***	0.023***	0.014***	0.021***
95%-CI	[0.064, 0.105]	[0.059, 0.070]	[0.062, 0.110]	[0.015, 0.030]	[0.012, 0.017]	[0.012, 0.029]
SE	(0.011)	(0.003)	(0.012)	(0.004)	(0.001)	(0.004)
p-value	(<0.001)	(<0.001)	(<0.001)	(<0.001)	(<0.001)	(<0.001)
GI-factor high (Ref. Category: GI-factor low)		-0.005	0.001		-0.004*	-0.002
95%-CI		[-0.014, 0.005]	[-0.010, 0.012]		[-0.007, -0.000]	[-0.006, 0.002]
SE		(0.005)	(0.006)		(0.002)	(0.002)
p-value		(0.317)	(0.867)		(0.034)	(0.334)
Ownership: public (Ref. Category: Ownership non-profit)	0.007*	0.007*	0.007*	0.002	0.001	0.001
95%-CI	[0.001, 0.014]	[0.001, 0.014]	[0.001, 0.014]	[-0.001, 0.004]	[-0.001, 0.004]	[-0.001, 0.004]
SE	(0.003)	(0.003)	(0.003)	(0.001)	(0.001)	(0.001)
p-value	(0.020)	(0.021)	(0.020)	(0.189)	(0.221)	(0.216)
Ownership: private (Ref. Category: Ownership non-profit)	-0.001	-0.001	-0.001	0.002	0.002	0.002
95%-CI	[-0.010, 0.008]	[-0.009, 0.008]	[-0.010, 0.008]	[-0.002, 0.005]	[-0.001, 0.005]	[-0.002, 0.005]
SE	(0.004)	(0.004)	(0.004)	(0.002)	(0.002)	(0.002)
p-value	(0.820)	(0.872)	(0.821)	(0.309)	(0.281)	(0.311)
Number of beds (by 100)	-0.003***	-0.003***	-0.003***	-0.000	-0.000	-0.000
95%-CI	[-0.004, -0.002]	[-0.004, -0.002]	[-0.004, -0.002]	[-0.001, 0.000]	[-0.001, 0.000]	[-0.001, 0.000]
SE	(0.001)	(0.001)	(0.001)	(0.000)	(0.000)	(0.000)
p-value	(<0.001)	(<0.001)	(<0.001)	(0.750)	(0.790)	(0.736)
Teaching status: academic teaching hospital (Ref. Category: Teaching status: no teaching assignment)	-0.007*	-0.007*	-0.007*	-0.001	-0.001	-0.001
95%-CI	[-0.014, -0.000]	[-0.014, -0.000]	[-0.014, -0.000]	[-0.004, 0.001]	[-0.004, 0.001]	[-0.004, 0.001]
SE	(0.004)	(0.004)	(0.004)	(0.001)	(0.001)	(0.001)
p-value	(0.037)	(0.042)	(0.037)	(0.263)	(0.324)	(0.305)
Teaching status: university hospital (Ref. Category: Teaching status: no teaching assignment)	0.018+	0.018+	0.018+	0.010**	0.010**	0.010**
95%-CI	[-0.001, 0.038]	[-0.001, 0.038]	[-0.001, 0.038]	[0.002, 0.017]	[0.003, 0.017]	[0.002, 0.017]
SE	(0.010)	(0.010)	(0.010)	(0.004)	(0.004)	(0.004)
p-value	(0.068)	(0.066)	(0.069)	(0.009)	(0.008)	(0.008)
Transformational leadership	-0.002		-0.003	-0.001		-0.001
95%-CI	[-0.009, 0.004]		[-0.009, 0.004]	[-0.003, 0.001]		[-0.003, 0.002]
SE	(0.003)		(0.003)	(0.001)		(0.001)
p-value	(0.446)		(0.436)	(0.410)		(0.582)
Social capital	-0.004		-0.004	-0.002		-0.001
95%-CI	[-0.011, 0.003]		[-0.012, 0.004]	[-0.004, 0.001]		[-0.004, 0.002]
SE	(0.004)		(0.004)	(0.001)		(0.001)
p-value	(0.283)		(0.285)	(0.194)		(0.357)
Num.Obs.	508	508	508	508	508	508
R2	0.085	0.079	0.085	0.047	0.045	0.049
R2 Adj.	0.072	0.068	0.070	0.034	0.034	0.034
AIC	-2056.9	-2055.6	-2054.9	-3076.6	-3077.4	-3075.5
BIC	-2018.8	-2021.8	-2012.6	-3038.5	-3043.6	-3033.2

+ p < 0.1, * p < 0.05, ** p < 0.01, *** p < 0.001

## Appendix 5

**Table:** Linear regression models (between-effects estimator) on the share of categories D and S (quality irregularities without category H) (n = 508)

	Model I:Quality irregularities (base model)	Model II:Quality irregularities (GI model)	Model III:Quality irregularities (GI model controlled)
Intercept	0.016***	0.015***	0.012*
95%-CI	[0.009, 0.024]	[0.013, 0.018]	[0.002, 0.023]
SE	(0.004)	(0.001)	(0.005)
p-value	(<0.001)	(<0.001)	(0.015)
GI-factor high (Ref. Category: GI-factor low)		-0.001	-0.002
95%-CI		[-0.003, 0.001]	[-0.005, 0.001]
SE		(0.001)	(0.002)
p-value		(0.235)	(0.213)
Ownership: public (Ref. Category: Ownership non-profit)	-0.003**	-0.003**	-0.003**
95%-CI	[-0.006, -0.001]	[-0.006, -0.001]	[-0.006, -0.001]
SE	(0.001)	(0.001)	(0.001)
p-value	(0.006)	(0.006)	(0.006)
Ownership: private (Ref. Category: Ownership non-profit)	-0.002	-0.002	-0.002
95%-CI	[-0.005, 0.002]	[-0.005, 0.002]	[-0.005, 0.002]
SE	(0.002)	(0.002)	(0.002)
p-value	(0.326)	(0.318)	(0.307)
Number of beds (by 100)	-0.000	-0.000	-0.000
95%-CI	[-0.001, 0.000]	[-0.001, 0.000]	[-0.001, 0.000]
SE	(0.000)	(0.000)	(0.000)
p-value	(0.267)	(0.264)	(0.257)
Teaching status: academic teaching hospital (Ref. Category: Teaching status: no teaching assignment)	-0.001	-0.001	-0.001
95%-CI	[-0.003, 0.002]	[-0.003, 0.002]	[-0.003, 0.002]
SE	(0.001)	(0.001)	(0.001)
p-value	(0.630)	(0.647)	(0.642)
Teaching status: university hospital (Ref. Category: Teaching status: no teaching assignment)	0.019***	0.019***	0.019***
95%-CI	[0.011, 0.026]	[0.011, 0.026]	[0.011, 0.027]
SE	(0.004)	(0.004)	(0.004)
p-value	(<0.001)	(<0.001)	(<0.001)
Transformational leadership	0.000		0.001
95%-CI	[-0.002, 0.002]		[-0.002, 0.003]
SE	(0.001)		(0.001)
p-value	(0.952)		(0.538)
Social capital	-0.001		0.000
95%-CI	[-0.003, 0.002]		[-0.003, 0.003]
SE	(0.001)		(0.002)
p-value	(0.642)		(0.972)
Num.Obs.	508	508	508
R2	0.071	0.073	0.074
R2 Adj.	0.058	0.062	0.059
AIC	-3034.7	-3037.8	-3034.3
BIC	-2996.6	-3004.0	-2992.0

+ p < 0.1, * p < 0.05, ** p < 0.01, *** p < 0.001
